# Protocol: Filgotinib in addition to methotrexate versus filgotinib monotherapy in patients with rheumatoid arthritis with an inadequate response to methotrexate: A multicenter, prospective, open-label, randomized controlled trial (FAITHFUL Study)

**DOI:** 10.1371/journal.pone.0322621

**Published:** 2025-05-19

**Authors:** Mitsuhiro Akiyama, Toshihiko Hidaka, Motohide Kaneko, Satoshi Ito, Hiroaki Taguchi, Tomonori Ishii, Shuji Asai, Shintaro Hirata, Kei Ikeda, Katsuya Suzuki, Naoki Kato, Yuko Kaneko

**Affiliations:** 1 Division of Rheumatology, Department of Internal Medicine, Keio University School of Medicine, Tokyo, Japan; 2 Institute of Rheumatology, Miyazaki Zenjinkai Hospital, Miyazaki, Japan; 3 Kaneko Internal Medicine Rheumatology Clinic, Kawaguchi, Japan; 4 Department of Rheumatology, Niigata Rheumatic Center, Shibata, Japan; 5 Department of Internal Medicine and Center for Arthritis and Rheumatic Disease, Kawasaki Municipal Kawasaki Hospital, Kawasaki, Japan; 6 Tohoku Medical and Pharmaceutical University, Division of Hematology and Rheumatology, Sendai, Japan; 7 Department of Orthopedic Surgery and Rheumatology, Nagoya University Graduate School of Medicine, Nagoya, Aichi, Japan; 8 Department of Clinical Immunology and Rheumatology, Hiroshima University Hospital, Hiroshima, Japan; 9 Department of Rheumatology, Dokkyo Medical University, Mibu, Japan; 10 Division of Rheumatology, Department of Internal Medicine, National Hospital Organization Tokyo Medical Center, Tokyo, Japan; 11 Translational Research Center for Medical Innovation, Foundation for Biomedical Research and Innovation, Kobe, Japan; Kyoto University Graduate school, JAPAN

## Abstract

**Background:**

Filgotinib (FIL), a Janus kinase-1 preferential inhibitor, has been studied for its efficacy and safety in rheumatoid arthritis. The FINCH3 trial compared FIL monotherapy, FIL plus methotrexate (MTX) combination therapy, and MTX monotherapy in MTX-naïve patients. However, comparisons in patients with an inadequate response to MTX remain unclear. This study aims to evaluate the efficacy and safety of FIL plus MTX versus FIL monotherapy in patients with rheumatoid arthritis who have an inadequate response to MTX.

**Methods and analysis:**

FAITHFUL (**F**ilgotinib **A**dd-on versus sw**IT**c**H** to **F**ilgotinib in patients with rhe**U**matoid arthritis who inadequate**L**y responded to methotrexate) study is a phase IV multicenter, prospective, open-label, randomized controlled trial. Patients with a history of inadequate response to at least 8 weeks of MTX and moderate or high disease activity will be assessed for eligibility at 10 centers in Japan. A history of Janus kinase inhibitor use is an exclusion criterion, but prior use of biologic agents is not considered. Enrolled patients will be randomly assigned in a 1:1 ratio to either the group adding FIL (Add-on group) or the group switching to FIL monotherapy (Switch group). The target sample size is 120 participants. The primary endpoint is the change in DAS28-CRP from baseline to week 24, aiming to assess if the Switch group is non-inferior to the Add-on group. Safety will be evaluated by assessing the incidence of adverse events.

**Ethics and dissemination:**

The study has received approval from the Certified Review Board of Keio University Hospital (N20230002) and adheres to the principles outlined in the Declaration of Helsinki and good clinical practice standards. Prior to enrollment, all participants provide written informed consent. The findings from this study are intended to be submitted for publication in relevant peer-reviewed journals.

**Trial registration:**

The trial was registered at Japan Registry of Clinical Trials (jRCTs031230673).

## Introduction

Rheumatoid arthritis is an autoimmune disease that primarily affects the joints, which can result in joint pain, swelling, stiffness and, eventually, joint damage and deformities [1,2]. The prevalence of rheumatoid arthritis is estimated to be around 1% worldwide [1]. The peak onset of rheumatoid arthritis typically occurs in individuals in their 40s and 50s, an age associated with peak social productivity. This can lead not only to physical disability but also to significant social limitations, economic burden, and psychological distress. If left untreated, approximately 70% of patients will develop bone erosions within 2 years [3]. Therefore, early and effective treatment intervention is fundamentally essential.

Disease-modifying antirheumatic drugs (DMARDs) are used to improve the clinical symptoms and prevent joint damage in patients with rheumatoid arthritis. The 2022 update of European League Against Rheumatism (EULAR) recommendations suggest that, unless contraindicated, treatment should begin with methotrexate (MTX), aiming to achieve clinical remission or at least low disease activity within 3–6 months [4]. If this goal is not reached, the addition of biological DMARDs (bDMARDs) or targeted synthetic DMARDs is recommended [4].

Filgotinib, a Janus kinase-1 preferential inhibitor, is one of the targeted synthetic DMARDs and has been evaluated for its efficacy and safety in the treatment of rheumatoid arthritis. The DARWIN1 and DARWIN2 studies were phase II trials that investigated the combination therapy of filgotinib and MTX, and the efficacy of filgotinib monotherapy, respectively, in patients with rheumatoid arthritis with an inadequate response to MTX [5,6]. Both studies showed positive results in the primary endpoint, which was the American College of Rheumatology (ACR) 20 improvement rate at 12 weeks. However, neither trial directly compared the combination therapy of filgotinib and MTX with filgotinib monotherapy. The FINCH1 trial was phase III study that assessed the efficacy and safety of the combination therapy of filgotinib and MTX in patients with moderate to severe active rheumatoid arthritis who had an inadequate response to MTX [7]. The FINCH2 trial evaluated the efficacy and safety of the combination therapy of filgotinib and conventional synthetic DMARDs (csDMARDs) in patients with moderate to severe active rheumatoid arthritis who had an inadequate response or were intolerant to biologic agents [8]. Both the FINCH1 and FINCH2 trials did not directly compare the combination therapy of filgotinib and MTX with filgotinib monotherapy. In the FINCH3 trial, the combination of filgotinib and MTX was compared to filgotinib monotherapy [9]. However, the trial included patients who had never been treated with MTX, so it did not directly compare the therapies in patients who had not responded to standard treatments, such as MTX or MTX combined with bDMARDs. Thus, it remains unknown whether filgotinib monotherapy and the combination of filgotinib and MTX are equally effective and safe in patients with an inadequate response to MTX.

Randomized controlled trials comparing the combination of tocilizumab and MTX with a switch to tocilizumab monotherapy in rheumatoid arthritis patients with an inadequate response to MTX have reported a higher incidence of liver dysfunction in the combination therapy group [10,11]. Additionally, long-term MTX use is associated with various complications, such as hepatotoxicity, bone marrow suppression, and lymphoproliferative disorders. Given that filgotinib is effective even without MTX, filgotinib monotherapy is expected to offer a safer treatment option while maintaining efficacy in patients with rheumatoid arthritis who do not respond adequately to MTX. Therefore, we aim to compare the efficacy and safety of filgotinib monotherapy with the combination therapy of filgotinib and MTX in patients with moderate to severe active rheumatoid arthritis who had an inadequate response to MTX.

## Materials and methods

### Trial design

**Fig 1:**
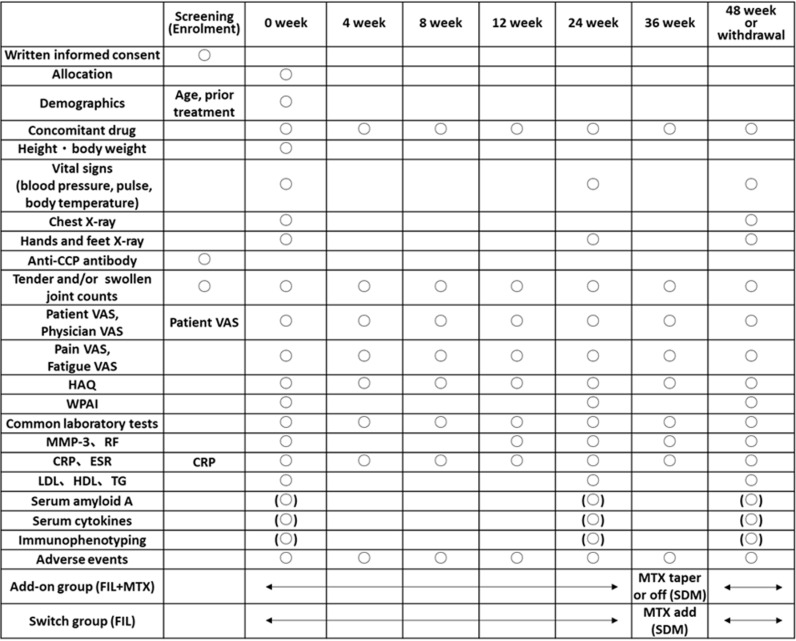
SPIRIT schedule. Common laboratory tests include white blood cell count, white blood cell differential (eosinophils, basophils, neutrophils, lymphocytes, atypical lymphocytes, monocytes), red blood cell count, hemoglobin, mean corpuscular volume, platelet count, erythrocyte sedimentation rate, blood urea nitrogen, creatinine, uric acid, total bilirubin, aspartate aminotransferase, alanine aminotransferase, lactate dehydrogenase, gamma-glutamyl transferase, alkaline phosphatase, C-reactive protein, total protein, albumin, immunoglobulin G. Baseline X-ray examinations of the hands and feet can use results obtained within 12 weeks prior. X-ray examinations of the hands and feet are not required at the time of discontinuation if the discontinuation occurs within 28 days of the previous X-ray examination. Abbreviation: VAS: Visual Analog Scale; HAQ: Health Assessment Questionnaire; WPAI: Work Productivity and Activity Impairment; MMP-3: Matrix Metalloproteinase-3; RF: Rheumatoid Factor; CRP: C-reactive Protein; ESR: Erythrocyte Sedimentation Rate; LDL: Low-Density Lipoprotein; HDL: High-Density Lipoprotein; TG: Triglycerides; SDM: Shared Decision-Making; FIL: Filgotinib; MTX: Methotrexate.

**Fig 2:**
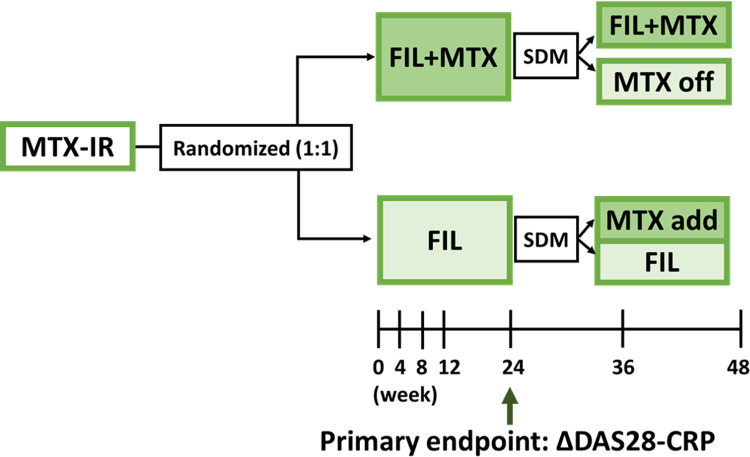
Study design. At 24 weeks, SDM determines whether to discontinue or initiate MTX. In the FIL + MTX group, MTX may be discontinued, whereas in the FIL group, MTX may be added if deemed necessary. Abbreviation: MTX-IR, methotrexate-inadequate response; FIL, filgotinib; SDM, shared decision making; DAS28-CRP, disease activity score 28-C-reactive protein.

FAITHFUL (**F**ilgotinib **A**dd-on versus sw**IT**c**H** to **F**ilgotinib in patients with rhe**U**matoid arthritis who inadequate**L**y responded to methotrexate) is a multicenter, open-label, randomized, controlled, investigator-initiated non-inferiority trial conducted at 10 rheumatology centers in Japan (Fig 1 and 2). Patients with rheumatoid arthritis who have an inadequate response to MTX or bDMARDs combined with MTX will be randomly assigned, in a 1:1 ratio, to either the group adding filgotinib (Add-on group) or the group switching to filgotinib monotherapy (Switch group). MTX will be maintained at the same dose as at enrollment unless clinically significant adverse events occur. bDMARDs and csDMARDs will be discontinued at the start of filgotinib administration. The primary endpoint is the change in disease activity score 28 (DAS28)-C-reactive protein (CRP) from baseline at week 24. After week 24, patients in the Add-on group may reduce or discontinue MTX if desired and agreed upon by the attending rheumatologist, while patients in the Switch group may resume MTX administration.

### Eligibility criteria

Eligible patients are those who satisfy all the inclusion criteria and do not meet any of the exclusion criteria.

### Inclusion criteria

1) Patients aged 18 years or older
2) Patients diagnosed with rheumatoid arthritis based on the 1987 ACR classification criteria or the 2010 ACR/EULAR classification criteria
3) Patients who have received stable treatment with MTX at a dose of at least 6 mg/week or combination therapy with bDMARDs and MTX for at least 8 weeks prior to enrollment but still demonstrate moderate to high disease activity, defined by a DAS28-CRP score of 3.2 or higher
4) Patients with at least 2 tender joints and at least 2 swollen joints
5) Patients who have not previously used JAK inhibitors
6) Patients may receive glucocorticoids at a dose equivalent to 10 mg/day or less of prednisolone
7) Patients who have provided written consent to participate in this study

### Exclusion criteria

1) Patients who plan to become pregnant or give birth between the time of consent and the completion of study treatment
2) Patients with an estimated glomerular filtration rate less than or equal to 30 mL/min/1.73 m²
3) Patients with aspartate aminotransferase or alanine aminotransferase levels exceeding five times the upper normal limit
4) Patients with a white blood cell count below 2000/μL
5) Patients with a platelet count below 100,000/μL
6) Patients with a history of venous thromboembolism within 2 months prior to obtaining consent
7) Patients with a history of acute interstitial pneumonia within 2 months prior to obtaining consent
8) Patients with a history of herpes zoster within 2 months prior to obtaining consent
9) Patients with a history of hypersensitivity to either filgotinib or MTX components
10) Patients who are breastfeeding
11) Patients with pleural effusion, ascites, or other similar conditions
12) Patients with active tuberculosis
13) Patients deemed unsuitable for participation in the study by the principal investigator or sub-investigator

### Withdrawal criteria

Subjects must be withdrawn from the study if they meet any of the withdrawal criteria listed below. Upon withdrawal, subjects should undergo the specified assessments and tests for early termination. Additionally, the reason for withdrawal should be explained to the subject as appropriate, and post-withdrawal treatment should be provided in good faith to ensure the subject experiences no disadvantage. The principal investigator or sub-investigator must document the date and reason for withdrawal in the electronic data capture (EDC) system. For traceable subjects, their clinical course should be monitored and reported to the Study Secretariat. Subjects withdrawn from the study will not be replaced, regardless of the reason for withdrawal.

1) Subject wishes to discontinue his/her study treatment
2) Subject wishes to withdraw his/her consent
3) Serious (or significant) adverse event occurs, and the study treatment is determined to be difficult to continue
4) A comorbidity (complication, etc.) exacerbates, and the study treatment is determined to be difficult to continue
5) If the study treatment is interrupted for more than 28 consecutive days
6) Confirmed pregnancy
7) If a significant protocol deviation is identified and it is determined that continuing the study would be inappropriate
8) If it is discovered after enrollment that the subject does not meet the inclusion criteria or violates the exclusion criteria, and it is determined that continuing the study would be inappropriate
9) If, for any other reason, the principal investigator or sub-investigator determines that continuing the study is not appropriate

### The status and timeline of the study

This trial was registered at Japan Registry of Clinical Trials (jRCTs031230673) in March 2024. Currently enrollment is ongoing.

Enrollment period: Publication date of the implementation plan – September 2025 (18 months).

Intervention period: 48 weeks

Data collection, analysis, and report generation periods: 2 years

First patient first visit: April 2024

Last patient first visit: September 2025

Last patient last visit: September 2026

Data collection and Fix: March 2027

Analysis and Clinical Study Report: September 2027

Total study period: 3 years and 6 months

### The details of each rheumatology center involved in this study and site-specific recruitment date

1. Division of Rheumatology, Department of Internal Medicine, Keio University School of Medicine, Tokyo, Japan. 25/April/2024
2. Institute of Rheumatology, Miyazaki Zenjinkai Hospital, Miyazaki, Japan. 03/July/2024
3. Kaneko Internal Medicine Rheumatology Clinic, Kawaguchi, Japan. 23/July/2024
4. Department of Rheumatology, Niigata Rheumatic Center, Shibata, Japan. 28/June/2024
5. Department of Internal Medicine and Center for Arthritis and Rheumatic Disease, Kawasaki Municipal Kawasaki Hospital, Kawasaki, Japan. 08/August/2024
6. Tohoku Medical and Pharmaceutical University, Division of Hematology and Rheumatology, Sendai, Japan. 05/September/2024
7. Department of Orthopedic Surgery and Rheumatology, Nagoya University Graduate School of Medicine, Nagoya, Aichi, Japan. 23/July/2024
8. Department of Clinical Immunology and Rheumatology, Hiroshima University Hospital, Hiroshima, Japan. 10/October/2024
9. Department of Rheumatology, Dokkyo Medical University, Mibu, Japan. 24/December/2024
10. Division of Rheumatology, Department of Internal Medicine, National Hospital Organization Tokyo Medical Center, Tokyo, Japan. 23/December/2024

### Allocation and blinding

A minimization method is used with history of bDMARDs use (yes/no) and anti-cyclic citrullinated peptide antibody status (positive/negative). Eligible patients will be randomly assigned electronically by a central system in a 1:1 ratio to either the combination therapy group (Add-on group) or the group switched to filgotinib monotherapy (Switch group). This is an open-label trial with no blinding implemented.

### Sample size calculation

Based on the results from the DARWIN1, DARWIN2, and FINCH3 trials, this study assumes a mean difference in change from baseline in DAS28-CRP at week 24 between the two groups of 0.07, with a standard deviation of 1.1. With an equal sample size ratio of 1:1 between the groups, and assuming a mean difference of 0.07, a standard deviation of 1.1, a one-sided significance level of 0.05, and a power of 80%, the required sample size was calculated to be 108 participants (54 per group). As for the sample size calculation, a one-sided test was employed based on the clinical assumption that filgotinib monotherapy would not be inferior to the combination of filgotinib and MTX. Considering a dropout rate of approximately 10%, the total sample size was set at 120 participants (60 per group).

### Trial treatments

#### Details of the study treatment.

Subjects who meet the inclusion criteria above and do not meet any exclusion criteria will initiate either a combination therapy with filgotinib 200 mg/day (or 100 mg/day if estimated glomerular filtration rate is below 60 mL/min/1.73 m²) and MTX (Add-on group) or filgotinib monotherapy (Switch group) after completing the baseline assessment (week 0). bDMARDs and csDMARDs other than MTX must be discontinued at the start of filgotinib administration. In the Add-on group, MTX should be maintained at the same dose as at enrollment until week 24 unless clinically significant adverse events occur. After week 24, patients in the Add-on group may reduce or discontinue MTX, while patients in the Switch group may resume MTX administration if desired by the patient and agreed upon by the attending rheumatologist.

#### Concomitant medications.

***Permitted concomitant therapies***

The use of oral glucocorticoids (≤10 mg/day in prednisolone equivalents), oral or suppository nonsteroidal anti-inflammatory drugs, and topical medications (skin ointments, eye drops, nasal sprays, inhaled medications) is permitted. Intravenous administration of glucocorticoids at a dose equivalent to ≤10 mg/day of prednisolone, or intra-articular injection of triamcinolone acetonide 40 mg, may be temporarily prescribed if oral administration is difficult or for other reasons. Additionally, glucocorticoid use for indications other than rheumatoid arthritis is permitted without dose restrictions, but only for a duration of up to 2 weeks.

#### Prohibited and restricted concomitant therapies.

Use of csDMARDs other than MTX, JAK inhibitors other than filgotinib, or bDMARDs is prohibited from the start of filgotinib administration until the end of the study treatment. Administration of live vaccines is also prohibited. For medications listed as requiring caution in the package insert (itraconazole, rifampin, famotidine, omeprazole, midazolam, metformin, ethinylestradiol, levonorgestrel, pravastatin, rosuvastatin, and atorvastatin), their use is permitted but should be conducted with careful monitoring of the patient’s condition.

### Outcomes

#### Primary endpoint.

The primary endpoint is the change in DAS28-CRP from baseline at week 24, with the objective of determining if the Switch group is non-inferior to the Add-on group. The non-inferiority margin of 0.6 was adopted based on the study by Rubbert-Roth et al [12]. The value of 0.6 was derived by Rubbert-Roth and colleagues based on the meta-analysis results of treatment effects using DAS28 in patients with rheumatoid arthritis, as well as the fact that a change of 0.6 or less is internationally adopted in the EULAR response criteria as a threshold indicating no clinically significant change [13].

#### Secondary endpoints.

1) Remission achievement rate

The rate of achieving remission at each time point based on DAS28-CRP, Simplified Disease Activity Index (SDAI), Clinical Disease Activity Index (CDAI), and Boolean criteria will be analyzed. The denominator for the proportion will be the number of full analysis set (FAS) in each group, and subjects who discontinue early will be considered as non-achievers. The definition of remission is as follows: DAS28-CRP < 2.6, SDAI ≤3.3, CDAI ≤2.8, Boolean criteria with ≤1 for all of the following items: tender joint count, swollen joint count, patient visual analog scale (VAS), and CRP.

2) Low disease activity achievement rate

The rate of achieving low disease activity at each time point based on DAS28-CRP, SDAI, and CDAI will be analyzed. The denominator for the proportion will be the number of FAS in each group, and subjects who discontinue early will be considered as non-achievers. The definition of achieving low disease activity is as follows: DAS28-CRP ≤ 3.2, SDAI≤11, and CDAI≤10.

3) Change from baseline in DAS28-CRP, SDAI, and CDAI

Change from baseline in DAS28-CRP, SDAI, and CDAI at each time point will be analyzed.

4) ACR20/50/70 and ACR-N achievement rates

Achievement rates of ACR20, ACR50, and ACR70, as well as ACR-N at each time point, will be analyzed. For ACR20, ACR50, and ACR70, the denominator for the proportion will be the number of FAS in each group, and subjects who discontinue early will be considered as non-achievers.

5) Functional remission achievement rate

The rate of achieving functional remission in Health Assessment Questionnaire (HAQ) at each time point will be analyzed. The denominator for the proportion will be the number of FAS in each group, and subjects who discontinue early will be considered as non-achievers. Functional remission is defined as HAQ ≤ 0.5.

6) Structural remission achievement rate

The rate of achieving structural remission in modified Total Sharp Score (mTSS) at week 24 and week 48 will be analyzed. The denominator for the proportion will be the number of FAS in each group, and subjects who discontinue early will be considered as non-achievers. Structural remission at both week 24 and week 48 is defined as an mTSS progression of ≤0.5.

7) Clinical relevant radiographic progression (CRRP, ΔmTSS ≥3) and rapid radiographic progression (RRP, ΔmTSS ≥5) rates

The rate of CRRP and RRP at week 24 and week 48 will be analyzed. The denominator for the proportion will be the number of FAS in each group, with the numerator for the CRRP rate being the number of cases with an mTSS increase of 3 or more at week 24 and 48, and the numerator for the RRP rate being the number of cases with an mTSS increase of 5 or more at week 24 and 48.

8) Cumulative values of CRP and matrix metalloproteinase (MMP)-3

The cumulative values of CRP and MMP-3 will be analyzed, with definition as the area under the curve (AUC) for CRP and MMP-3 for each subject.

9) Work productivity and activity impairment (WPAI)

WPAI at week 24 and week 48 will be analyzed. At week 24, summary statistics will be calculated for baseline values, measurements at each time point (week 24 and week 48), and changes from baseline in the two groups (Add-on group and Switch group). At week 48, the analysis will include four groups (Add-on to Switch group and Switch to Add-on group in addition to the original two groups).

#### Other efficacy endpoints.

Change from baseline in DAS28-erythrocyte sedimentation rate (ESR), tender joint count, swollen joint count, patient VAS, physician VAS, pain VAS, fatigue VAS, HAQ, CRP, ESR, MMP-3, rheumatoid factor (RF), and mTSS at each time point will be analyzed.

#### Safety endpoints.

Number of cases and incidence rate of adverse events, clinical laboratory values, and vital signs will be recorded from the first dose until week 48 of study treatment initiation. An adverse event is any undesirable symptom, sign, disease, or abnormal clinical laboratory value that occurs in a subject, regardless of whether there is a causal relationship with the study. Worsening of arthritis is considered an insufficient effect of the study treatment and will not be classified as an adverse event. The principal investigator and others will assess the severity of adverse events in three categories (mild, moderate, and severe). The severity of adverse events (mild, moderate, and severe) will be assessed based on the Common Terminology Criteria for Adverse Events (CTCAE). This assessment of severity is different from the evaluation of the seriousness of the adverse event. A serious adverse event is an adverse event that results in death, is life-threatening, requires hospitalization or prolongation of an existing hospitalization, leads to permanent or significant disability or dysfunction, or causes congenital abnormalities in offspring. On the other hand, hospitalizations without adverse events will not be considered serious adverse events if they fall under the following categories:

1) Hospitalizations for purposes other than the treatment or evaluation of an adverse event (e.g., health checkups).
2) Hospitalizations for scheduled surgeries or examinations planned before informed consent, provided there is no change in the patient’s condition after initiating the study treatment.
3) Hospitalizations required for the administration of the study drug.
4) Hospitalizations for the periodic maintenance of pre-existing medical devices (e.g., battery replacements).

Each investigator will make determinations based on these predefined criteria.

All adverse events must be followed up for 28 days after the completion of study treatment or until recovery, whichever comes first. Additionally, serious adverse events must be followed up until they resolve or, if resolution is not expected, until the symptoms stabilize. However, if it is medically determined that follow-up is not necessary, the follow-up for the adverse event may be discontinued.

#### Exploratory endpoints.

Change from baseline at week 24 and week 48 in serum amyloid A, serum cytokines, and peripheral blood immunophenotype will be measured. Specifically, serum cytokine levels will be measured using Olink proteomics and/or other methods. Blood lymphocyte subsets, including those defined by the Human Immunology Project, as well as T cell immunoreceptor with Ig and ITIM domains (TIGIT)-positive T follicular helper (Tfh) cells and C-X3-C motif chemokine receptor 1 (CX3CR1)-positive T cells, will be analyzed using flow cytometry and/or other methods [14–16]. Serum cytokine measurements and immunophenotyping will be conducted only at the rheumatology department of Keio University Hospital.

### Data collection

#### Trial visits and examinations.

The study visit schedule and data collection timeline are outlined in Fig 1. The screening period will be within 8 weeks prior to the start of the study treatment. The assessment time points will be counted from the start date of the study treatment (Day 1), with evaluations to be conducted within ±1 week of each time point (weeks 4–12), and within ±2 weeks of each time point (from week 24 onwards).

### Data management, monitoring, and auditing

At enrollment (within 8 weeks prior to the start of study treatment), the following information will be collected and recorded in the EDC system. In addition to this information, the registration number, status of informed consent, and results of the eligibility assessment will also be recorded in the EDC system:

1) Gender
2) Age (at the time of consent)
3) Race (Asian, American Indian or Alaska Native, Black or African American, Native Hawaiian or Other Pacific Islander, White)
4) Height and weight
5) Time of rheumatoid arthritis diagnosis
6) Treatment history of rheumatoid arthritis (glucocorticoids, DMARDs, NSAIDs, etc.)
7) Surgical history related to rheumatoid arthritis
8) Medical history and comorbidities
9) Smoking history
10) 12-lead electrocardiogram (results obtained within 12 weeks prior to consent can be used)
11) Infectious disease testing (results obtained within 12 weeks prior to consent can be used; for patients using bDMARDs, pre-treatment results can be used): Either skin tuberculin test or interferon-gamma release assay (IGRA), hepatitis B surface (HBs) antigen, HBs antibody, HB core antibody, and anti- hepatitis C virus antibody. For subjects who are HBs antigen-negative but HBs antibody-positive and/or HBc antibody-positive, HBV-DNA quantification testing must be performed at least once every 6 months as a minimum requirement. This does not preclude more frequent testing, such as every 1–3 months.

The case report forms for this study will be managed using the EDC system, and the registration numbers will be issued through the EDC system. The principal investigator, co-investigators, or research collaborators will promptly enter data into the EDC system after the evaluation/investigation of each participant is completed. In accordance with a separate data management plan, the data center will follow up on delayed data entries, conduct data inquiries, address queries related to the data, make necessary data corrections, manage the database, and prepare datasets for statistical analysis based on the data entered into the database. The principal investigator will ensure that data and records are properly stored for 5 years from the later of the date the study conclusion is reported or 3 years after the final publication of the study results.

Independent monitors will visit the study sites to examine the records, verify them against source documents, and engage with the investigators and site coordinator to observe and discuss the trial’s progress. They are responsible for ensuring compliance with the protocol and guidelines, as well as overseeing the completion of the electronic Case Report Form (eCRF) and other required documentation.

This study is a collaborative research project with Gilead Sciences, Inc., the manufacturer of JYSELECAⓇ (filgotinib). Gilead Sciences, Inc. will be involved in the preparation of the study protocol. However, Gilead Sciences, Inc. will not participate in data collection, data management, statistical analysis, or the evaluation of study results. Therefore, audits are not considered mandatory, and routine audits will not be conducted. The reliability of this study is ensured by the fact that it is not a clinical trial aimed at regulatory approval. Monitoring will be implemented to protect study participants, ensure ethical standards, adhere to clinical research laws and the study protocol, and maintain data reliability and appropriate research practices.

In this study, both central monitoring and on-site monitoring will be conducted. Central monitoring will be performed every six months in principle. It will include the review of progress management items (enrollment progress and status of discontinued cases), safety management items (compliance with the study protocol and the presence or absence of adverse events), and outcome-related items (the implementation status of the primary endpoint, secondary endpoints, and efficacy assessment items). On-site monitoring will be conducted twice: once within three months after the enrollment of the first patient at each site and once after treatment completion or after the enrollment of the last patient at each site. The monitoring will include confirmation of the informed consent process, eligibility of enrolled patients, and compliance with the study protocol with data accuracy.

The accuracy, consistency, and reliability of the data will be scrutinized through independent monitoring, which operates separately from the study investigators as noted above. Furthermore, a steering committee consisting of external experts, independent of the study investigators, will conduct a thorough review of the data.

Furthermore, when publishing papers or presenting at conferences, the conflict of interest (COI) and the role of Gilead Sciences, Inc. in this study will be clearly disclosed. Quality control will be conducted through monitoring, and if significant issues arise or if events with serious implications for participant safety occur, consideration will be given to conducting an ad-hoc audit as necessary.

### Analysis population

#### Safety analysis population.

All subjects who have received at least one dose of the study drug (filgotinib) will be included in the safety analysis population.

#### Full analysis set (FAS).

All subjects who have received the study drug (filgotinib) and have valid efficacy data with at least one measurable point will be included in the FAS, which will be used for efficacy analysis.

#### Per protocol set (PPS).

Among the FAS, subjects without any major protocol deviations will be included in the PPS as the population for sensitivity analysis of efficacy. The handling of major protocol deviations and cases will be determined through consultation between the lead investigator, data center, and statistical analysis team before finalizing the study data. In clinical trials, adherence rates for once-daily investigational drugs have generally been reported to be in the 70% range [17]. In our study, we adopted a slightly stricter criterion, defining non-adherence as an adherence rate of less than 80%. If the adherence rate to the study drug is less than 80% up to week 24, the subject will be excluded from the PPS analysis.

### Statistical methods

For the primary endpoint, the change in DAS28-CRP from baseline will be analyzed using a Mixed Effects Model for Repeated Measures (MMRM). This method considers the baseline measurement as a covariate and incorporates group, time point, and the interaction between time point and group as fixed effects. It also reflects the variance-covariance structure of repeated measurements within each patient. Using this model, the least squares mean difference between groups at week 24 and its 90% confidence interval will be calculated. Non-inferiority will be confirmed if the upper limit of this 90% confidence interval is less than 0.6.

For the secondary endpoints, the proportion of patients meeting each endpoint and the corresponding 95% confidence intervals will be calculated. For the change from baseline in each endpoint, summary statistics (number of patients, mean, standard deviation, minimum, median, and maximum) will be presented. Additionally, the change from baseline will be analyzed using the same MMRM approach as for the primary endpoint, estimating the least squares mean and its 95% confidence interval at each time point.

As a sensitivity analysis, stratification will be performed based on stratification variables (history of bDMARD use and anti-CCP antibody positivity/negativity), and summary statistics will be calculated for baseline values, measurements at each time point, and changes from baseline by treatment group and treatment period, with intergroup comparisons conducted using t-tests.

Additionally, as a supplementary analysis, in the PPS population, intergroup comparisons of changes from baseline will be conducted using MMRM, and summary statistics will be calculated with intergroup comparisons performed using t-tests. Furthermore, in the PPS population, stratification based on the aforementioned stratification variables will be conducted, and summary statistics will be calculated for baseline values, measurements at each time point, and changes from baseline by treatment group and treatment period, with intergroup comparisons conducted using t-tests.

For the cumulative values of CRP and MMP-3, the AUC for each participant will be calculated, and summary statistics (number of patients, mean, standard deviation, minimum, median, and maximum) for each group will be presented.

For the safety analysis, treatment-emergent adverse events (TEAEs) observed during the period from the start of the study treatment to the end of the observation period will be summarized. Adverse events that cannot be ruled out as being causally related to the study treatment will be classified as adverse drug reactions. For each group, the number and percentage of cases of TEAEs and adverse drug reactions will be summarized by system organ class and preferred term. Additionally, a summary by severity level will also be provided.

Missing data at each time point will not be imputed. For patients who discontinue the study, only the data obtained up to the point of discontinuation will be used for analysis. Additionally, when analyzing the proportion of patients who achieve the endpoint, cases with missing data at each time point, including those who discontinued the study, will be considered as non-responders and included in the denominator.

### Ethics and dissemination

#### Ethical approval.

The study has been approved by the Certified Review Board of Keio University Hospital (N20230002), and conforms to the Declaration of Helsinki and good clinical practice guidelines. Written informed consent is obtained from participants prior to enrollment. Any significant modifications to the study protocol must receive approval from the Certified Review Board of Keio University Hospital.

#### Informed consent.

The principal investigator and other study personnel will provide appropriate and sufficient explanations using written documents. The explanation documents will be written in simple language that the participants can understand, and will be used after obtaining the opinion of the Certified Review Board of Keio University Hospital and the approval of the institutional administrator. The documents will be appropriately version-controlled. The written and oral explanations will include the purpose, methods, anticipated outcomes, risks, and potential disadvantages of the study, as well as the fact that participation is voluntary, that refusal to participate will not result in any disadvantage, and that participants can withdraw their consent at any time.

Informed consent will be confirmed by the participant signing the consent document. The distribution and storage methods of the consent document will follow the procedures of each institution. If new information regarding efficacy, safety, or other aspects of the study that could affect the participant’s consent is obtained, or if there are changes to the study protocol that could influence consent, the participant will be promptly provided with updated information.

#### Confidentiality.

Data related to subjects enrolled in this study will be coded using the registration number issued at the time of enrollment and entered into the EDC system. Personal information that can directly identify subjects, such as names, addresses, or phone numbers, will not be recorded in the database. Furthermore, any information related to subjects’ privacy obtained during the study will not be disclosed to third parties by study personnel.

#### Dissemination plan.

The total study duration is expected to be 3 years and 6 months, with the study scheduled to conclude in September 2027. Data from all centers will be analyzed collectively and the results of this study are planned to be submitted for presentation in scientific conferences and publication in relevant peer-review journals.

### Study discontinuation criteria

The study will be discontinued if any of the following criteria are met. The principal investigator will promptly inform the principal investigators at each participating medical institution about the decision to discontinue the study. Each principal investigator will promptly notify the subjects and take appropriate medical interventions and other necessary actions.

1) If the number of enrolled subjects is significantly below the target, or if frequent deviations from the study protocol occur, making it difficult to complete the study
2) If a serious adverse event is reported and it is determined that there are safety concerns regarding the protocol treatment
3) If new information related to the protocol treatment is obtained, such as from publications or conference presentations, and it is determined that there are safety concerns regarding the protocol treatment

## Discussion

The FAITHFUL study protocol outlines a comprehensive investigation into the efficacy and safety of filgotinib monotherapy versus combination therapy with MTX in patients with rheumatoid arthritis who have shown inadequate response to MTX. Such direct comparisons are currently lacking in the literature, as acknowledged in the protocol. Therefore, this study design reflects current challenges in managing rheumatoid arthritis, such as balancing therapeutic efficacy and minimizing adverse effects.

In the FINCH3 trial, the only study comparing filgotinib plus MTX, filgotinib monotherapy, and MTX monotherapy in MTX-naive patients, adverse events up to week 24 were observed in 66.1% (275/416) of patients in the filgotinib 200 mg plus MTX group, 69.6% (144/207) in the filgotinib 100 mg plus MTX group, 55.7% (117/210) in the filgotinib 200 mg monotherapy group, and 63.2% (263/416) in the MTX monotherapy group [9]. Although no statistical tests were conducted, there was a tendency for higher rates in the filgotinib plus MTX groups compared to the filgotinib monotherapy group. Among the major adverse events, the frequency of nausea was 10.3% (43/416) in the filgotinib 200 mg plus MTX group, 15.5% (32/207) in the filgotinib 100 mg plus MTX group, 6.2% (13/210) in the filgotinib 200 mg monotherapy group, and 10.8% (45/416) in the MTX monotherapy group, indicating a trend toward higher rates in the MTX combination groups. Thus, if the FAITHFUL study demonstrates the non-inferiority of filgotinib monotherapy compared to filgotinib plus MTX combination therapy, it could serve as evidence for a treatment approach with equivalent efficacy and fewer adverse effects in the future. This would contribute to establishing a more appropriate treatment strategy for patients with rheumatoid arthritis with insufficient response to MTX.

The study protocol defines multiple efficacy endpoints, including DAS28-CRP changes, ACR response rates, quality-of-life measures such as HAQ and WPAI, and radiological changes, indicating a comprehensive endpoint design. Safety endpoints are also robust, emphasizing AEs and laboratory abnormalities. Furthermore, allowing shared decision-making between patients and rheumatologists post-week 24 reflects a patient-centric approach, providing flexibility in tapering or restarting MTX based on clinical need. On the other hand, the limitation of this study is the lack of blinding which may introduce bias in subjective endpoints like patient-reported outcomes. However, this open-label design allows for a more practical evaluation of treatment effectiveness in a real-world setting, where patients and physicians are aware of the treatments being administered, potentially enhancing adherence and capturing a broader range of patient experiences.

In clinical trials, outcome measures are often repeatedly assessed in the same individuals over time, resulting in longitudinal data. However, it is common for participants to discontinue the study or miss scheduled visits, leading to incomplete or missing data. MMRM is a likelihood-based approach that provides valid estimates under the assumption of missing at random (MAR), without requiring data imputation. MMRM utilizes available data while accounting for baseline covariates and within-subject correlations, making it a robust method when the mean and covariance structures are correctly specified. However, as the missing data mechanism cannot be directly verified from observed data, and MMRM is only valid under MAR, it is important to consider alternative analytical approaches when its assumptions are not met. In this study, we acknowledge these limitations and will explore additional sensitivity analyses to ensure the robustness of our findings.

In conclusion, the FAITHFUL study protocol is well-designed to address an important clinical question in the management of rheumatoid arthritis. By leveraging a randomized, multicenter approach, the study could provide pivotal evidence on the viability of filgotinib monotherapy as an effective alternative for MTX-refractory patients.
